# Consanguinity and Diabetes in Saudi Population: A Case-Control Study

**DOI:** 10.7759/cureus.20836

**Published:** 2021-12-30

**Authors:** Saad H Alzahrani, Nawaf M Alzahrani, Fahad M Al Jabir, Mohammed K Alsharef, Shawana Zaheer, Salma H Hussein, Abdullah M Alguwaihes, Anwar A Jammah

**Affiliations:** 1 Obesity and Endocrine Metabolism Center, King Fahad Medical City, Riyadh, SAU; 2 College of Medicine, King Saud Bin Abdulaziz University for Health Sciences, Riyadh, SAU; 3 Endocrinology, Security Forces Hospital, Riyadh, SAU; 4 Endocrinology Division, Department of Medicine, King Saud University Medical City, Riyadh, SAU

**Keywords:** saudi arabia, patterns, family history, diabetes mellitus, consanguinity

## Abstract

Background and Aim: Diabetes mellitus (DM) of both types is a genetically determined disorder and is prevalent in the Saudi population. Furthermore, the rate of consanguineous marriages is also high among Saudis. Therefore, we aimed to determine the prevalence of consanguinity among people with DM and investigate the effect of consanguinity on the occurrence of diabetes at different levels.

Methods: A descriptive cross-sectional study was carried out at the Obesity, Metabolism and Endocrine Center of King Fahad Medical City in Riyadh, Saudi Arabia in January 2021. Information on patients’ demographics (age, gender), family history of DM, and presence of consanguinity, and degree of consanguineous marriage were collected.

Results: We included 324 people with DM, 143 (44.1%) with type 1 diabetes (T1DM), and 181 (55.9%) with type 2 diabetes (T2DM). We included 201 people without DM for T1DM control and 300 people for T2DM control. The mean age was 26.6 ± 11.1 years for the T1DM group and 57.8 ± 11.6 years for the T2DM group. Consanguinity was noted among 73 (51.4%) T1DM patients, but T1DM was not significantly related to consanguinity. T2DM was significantly correlated with consanguinity (r=0.132, p=0.004) particularly among patients with a degree of consanguinity as first-cousins for both paternal and maternal sides (odds ratio [OR]=1.151 and 1.476). Gender and positive family history for DM and consanguineous marriage between cousins were significant factors for T2DM. After controlling for gender and a positive family history of DM, consanguineous marriage between cousins from both the paternal and maternal sides remained significant.

Conclusion: T2DM occurrence increases in presence of consanguinity in the Saudi population. This relationship might contribute to the higher risk of DM prevalence. Further studies are needed to elucidate this relationship deeply. It’s unclear whether lowering consanguineous marriages would decrease the prevalence of diabetes or not. However, a clear message about this correlation has to be delivered to the public.

## Introduction

Diabetes mellitus (DM) is a group of metabolic disorders characterized by hyperglycemia resulting from defects in insulin secretion, insulin action, or both. Type 1 diabetes (T1DM) accounts for 5-10% of those with diabetes, whereas type 2 diabetes (T2DM) accounts for ∼90-95% [1]. T1DM is often associated with a strong genetic predisposition, however, the genetics of this form of diabetes are complex, diverse, and not clearly defined [1-4].

There is a high rate of consanguinity in Saudi Arabia. In many Arab countries, consanguinity, endogamy, and first-cousin marriage rates are high. Because of this, the high possibility of increased homozygosity in the human leukocyte antigen (HLA) haplotypes and non-HLA genes from both sides increases their susceptibility to T1DM [5]. Furthermore, a family history of T2DM and parental history of consanguinity were found to increase the risk of impaired fasting glucose and DM in the offspring [6].

Several studies on consanguinity and DM have been conducted in Saudi Arabia and other countries. ElMouzan et al. in 2008 found no significant association between first-cousin consanguinity and T1DM [7]. On the other hand, Gosadi et al. in 2014 found a significant correlation between increased fasting blood glucose (FBG) and increased degree of consanguinity, and that consanguinity increases the risk of T2DM by an earlier onset of the disease and by strengthening possible genetic effects on FBG [8]. A very recent article reported that parental consanguinity was not clearly linked to T1DM in children, however, a history of first-cousin parents increases the risk for T1DM [9]. Of note, maternal history of DM was associated with more DM in the offspring compared to the paternal history of DM [10]. A synergistic effect of familial aggregation and consanguinity in the Saudi population was previously observed [11].

This study was conducted to determine the prevalence of consanguinity among people with DM, investigate the effect of consanguinity and the degree of consanguinity on the occurrence of DM, and the effect of consanguinity in the presence of a positive family history of DM.

## Materials and methods

A descriptive cross-sectional study was carried out at the Obesity, Metabolism and Endocrine Center of King Fahad Medical City in Riyadh, Saudi Arabia in January 2021. People with DM were recruited from the Diabetes Center of King Fahad Medical City, Riyadh, Saudi Arabia. This study was approved by the Institutional Review Board of King Fahad Medical City, Riyadh, Saudi Arabia (approval number 17-042). Informed consent was obtained from all patients prior to the conduct of the study. All collected data were kept confidential and were used for research purposes only.

The sample size was calculated using the formula n = Z_α/2_^2^ ­*p*(1-p) / MOE^2^, where Z_α/2 _is the critical value of the normal distribution at α/2 (e.g. for a confidence level of 95%, α is 0.05 and the critical value is 1.96), MOE is the margin of error, p is the sample proportion, and N is the population size (a Finite Population Correction is applied to the sample size formula). The calculated sample size was 130 for T1DM and 170 for T2DM. It was decided to recruit 300 people with DM of both types. We recruited 300 people without DM as a control group.

Data collection was undertaken using a self-administered personally-delivered questionnaire in the Arabic language. Information on patients’ demographics (age, gender), family history of DM, and presence of consanguinity. Consanguinity was further detailed to which level according to the degree of consanguinity. DM in the control group was ruled out by measuring hemoglobin (Hb) A1c levels and excluding those above 5.6% (38 mmol/mol). All collected data were entered into a Microsoft Excel worksheet and data analysis was undertaken using the Statistical Package for Social Sciences (SPSS) version 26.0 (IBM Corp., Armonk, NY). Results were expressed as numbers and percentages for categorical variables and as mean and standard deviation for continuous variables such as age, body mass index (BMI), and HbA1c level. Correlations between consanguinity and variables were undertaken using the Pearson correlation test. Testing for any differences between the presence of consanguinity in either T1DM or T2DM, and proportions between two variables was undertaken using the Chi-square test (categorical variables) and Independent t-test (for continuous variables). A p-value of <0.05 was considered statistically significant.

## Results

We included 324 people with DM, of whom 143 (44.1%) had T1DM and 181 (55.9%) had T2DM. We also included 200 people without DM as the control for the T1DM group and 300 people for the T2DM group. The mean age for the T1DM patients was 26.6 ± 11.1 years, while the T2DM patients had a mean age of 57.8 ± 11.6 years. Compared to the control group, 55.6% of the T1DM group had a positive family history of DM (p=0.031). Patients with T2DM were predominantly females and 71.7% had a positive family history of DM.

Patients with type 1 diabetes mellitus

No significant difference in the mean BMI was observed between T1DM patients and controls (25.6 ± 5.5 versus 25.0 ± 2.7 kg/m^2^, p=0.172). Half of the T1DM patients (50.6%) had a maternal (29.1%) and paternal (21.5%) history of DM of both types (p=0.016). Consanguinity was noted among 73 (51.4%) people in the T1DM group, however, there was no significant difference in the proportion of consanguineous marriage among T1DM patients and controls (p=0.078) (Table 1).

**Table 1:** Demographic profile and consanguinity of T1DM patients and control T1DM = Type 1 diabetes mellitus; OR = Odds ratio; CI = Confidence interval; BMI = Body mass index; DM = Diabetes mellitus

Variables	No (%) of subjects		OR (95%CI)	p-values
	Type 1 DM	Controls		
	N=143	N=200		
Age group				
<35	110 (76.9)	200 (100)	1	
35 – 50	26 (18.2)	0	0.369 (0.320-0.426)	<0.001
>50	7 (4.9)	0	0.405 (0.356-0.461)	0.002
BMI in kg/m^2^				
<25 (normal)	77 (53.8)	100 (50.0)	1	
25-30 (overweight)	40 (28.0)	100 (50.0)	0.690 (0.579-0.821)	<0.001
>30 (obese)	26 (18.2)	0	0.369 (0.320-0.426)	<0.001
Gender				
Male	53 (37.1)	49 (24.5)	1	0.012
Female	90 (62.9)	151 (75.5)	0.767 (0.613-0.959)	
Family History of DM				
Yes	80 (55.9)	87 (43.5)	1	0.023
No	63 (44.1)	113 (56.5)	1.338 (1.039-1.724)	
Family History type				
Father	18(22.5)	13 (14.9)	1	
Mother	23 (28.8)	14 (16.1)	0.606 (0.464-0.792)	0.005
Sister	9 (11.3)	16 (18.4)	0.705 (0.513-0.969)	0.062
Brother	6 (7.5)	21 (24.1)	0.781 (0.558-1.094)	0.278
Both Parents	11 (13.8)	16 (18.4)	0.856 (0.601-1.218)	0.529
Grandparents	9 (11.3)	6 (6.9)	0.779 (0.498-1.217)	0.419
All Family Members	4 (5.0)	1 (1.1)	0.586 (0.367-0.936)	0.195
Consanguinity				
Yes	73 (51.0)	84 (42.0)	1	0.097
No	70 (49.0%)	116 (58.0)	0.858	
Consanguinity type				
1^st^ cousin paternal	25 (34.2)	44 (52.4)	1	
1^st^ cousin maternal	14 (19.2)	10 (11.9)	0.571 (0.422-0.772)	0.007
2^nd^/3^rd^ cousins	12 (16.4)	12 (14.3)	0.693 (0.482-0.997)	0.131
Common 4^th^-7^th^ grandparents	6 (8.2)	11 (13.1)	0.821 (0.539-1.251)	0.4
Not related but same tribe	16 (21.9)	7 (8.3)	1.191 (0.618-2.295)	0.625

Patients with type 2 diabetes mellitus

People in the T2DM group were significantly older than their non-diabetes controls (57.8 ± 11.6 versus 48.5 ± 6.0, p<0.001). BMI was significantly lower among T2DM patients compared to controls (31.0 ± 6.3 versus 34.3 ± 3.0, p<0.001). Positive family history of DM was documented in 147 (81.2%) of T2DM patients compared to (66.0%) of the non-diabetes control (p<0.001). There were 32 (21.9%) people in the T2DM group who had a positive family history of DM from both parents. Compared to controls, a significant proportion of the T2DM group had a father with DM (32.9%) or either one of the parents had DM (21.9% versus 18.7%%), p<0.001. The proportion of positive consanguinity was significantly greater among T2DM patients compared to controls (56.9% versus 43.3%, p=0.004) regardless of the degree of consanguinity (p=0.768) (Table 2).

**Table 2:** Demographic profile and consanguinity among T2DM patients and control T2DM = Type 2 diabetes mellitus; OR = Odds ratio; CI = Confidence interval; BMI = Body mass index; DM = Diabetes mellitus

Variables	No (%) of subjects		OR (95%CI)	p-values
	Type 2 DM	Controls		
	N=181	N=300		
Age group				
<35	4 (2.2)	0	1	
35-50	45 (24.9)	197 (65.7)	0.529 (0.452-0.620)	<0.001
>50	132 (72.9)	103 (34.3)	1.827 (1.561-2.139)	<0.0010
BMI in kg/m^2^				
<25 (normal)	28 (16.4)	0	1	
25-30 (overweight)	53 (31.0)	0	0.282 (0.242-0.329)	<0.001
>30 (obese)	90 (52.6)	300 (100)	4.333 (3.615-5.194)	<0.001
Gender				
Male	79 (43.6)	67 (22.3)	1	<0.001
Female	102 (56.4)	233 (77.7)	1.777 (1.426-2.215)	
Family History of DM				
Yes	147 (81.2)	198 (66.0)	1	<0.001
No	34 (18.8)	102 (34.0)	1.704 (1.243-2.337)	
Family History type				
Father	48 (32.9%)	37 (18.7%)	1	
Mother	21 (14.4%)	53 (26.8%)	2.176 (1.244-3.806)	0.008
Sister	9 (6.2%)	32 (16.2%)	2.060 (1.141-3.717)	0.005
Brother	15 (10.3%)	36 (18.2%)	1.941 (1.018-3.698)	0.046
Both Parents	32 (21.9%)	37 (18.7%)	0.819 (0.482-1.392)	0.497
Grandparents	1 (0.7%)	1 (0.5%)	0.446 (0.364-0.547)	0.165
All Family Members	20 (13.7%)	2 (1.0%)	0.092 (0.027-0.313)	<0.001
Consanguinity				
Yes	103 (56.9)	130 (43.3)	1	0.005
No	78 (43.1)	170 (56.7)	1.727 (1.190-2.506)	
Consanguinity type				
1^st^ cousin paternal	40 (38.8)	50 (38.5)	1	
1^st^ cousin maternal	12 (11.7)	9 (6.9)	0.436 (0.180-1.055)	0.059
2^nd^/3^rd^ cousins	14 (13.6)	21 (16.2)	0.898 (0.445-1.813)	0.764
Common 4^th^-7^th^ grandparents	6 (5.8)	9 (6.9)	0.902 (0.316-2.577)	0.847
Not related but same tribe	31 (30.1)	41 (31.5)	0.766 (0.461-1.273)	0.303

Correlations between DM and consanguinity and other variables

Among patients with T1DM, significant correlations were seen between DM and gender (r=0.127, p=0.019) and family history of DM (r=0.117, p=0.031). T1DM was not significantly related with consanguinity (p=0.079) and with the degree of consanguinity whether it was paternal, maternal, cousins, or grandparents (p=0.331, p=0.081, p=0.377, p=0.601 respectively). However, T1DM was negatively correlated with having DM of people from the same tribe but not blood related (r=-0.153, p=0.004).

Among patients with T2DM, significant correlations were seen between DM and gender (r=0.225, p<0.001) and family history of DM (r=0.164, p<0.001). T2DM was significantly correlated with consanguinity (r=0.132, p=0.004) particularly among patients with degree of consanguinity as cousins for both paternal and maternal side (r= 0.103, p=0.025, and r=0.134, p=0.003). The OR for consanguineous marriage between cousins from the paternal side was 1.151 (95%CI of 0.943 to 1.405), and from the maternal side was 1.476 (95% CI of 0.896 to 2.431).

Regression analysis of factors associated with T2DM

A logistic regression model was constructed for factors that were associated with DM as the dependent variable and gender, family history of DM, and consanguinity as independent variables. Gender and a positive family history for DM were significant factors (Beta=0.210, 95%CI=0.129 to 0.313, p<0.000 and Beta=0.142, 95% CI=0.059 to 0.246, p=0.001, respectively) and consanguineous marriage between cousins from both paternal and maternal side were also significant (Beta=0.105, 95%CI=0.247 to 0.013, p=0.029 and Beta=0.108, 95%CI =0.472 to 0.042, p=0.019). After controlling for gender and a positive family history of DM, consanguineous marriage between cousins from both the paternal and maternal side remained significant (Beta=0.094, 95%CI=0.230 to 0.004, p=0.043 and Beta=0.094, 95%CI=0.453 to 0.034, p=0.023).

## Discussion

This study aimed to investigate on the effect of consanguinity and the degree of consanguinity on the occurrence of DM, and the effect of consanguinity in the presence of positive family history of diabetes. This is the first case control study in Saudi Arabia that examines the relationship between consanguinity and DM in both genders. Consanguineous marriage in the Arab countries was reported to constitute 20-50% of all marriages, and this has been a subject in a multitude of controversial research because of the potential adverse health outcomes on offspring [12,13]. In Saudi Arabia, the rate of consanguineous marriage was 52.0% with first-cousin marriages being the commonest at 39.3% [14].

This study showed that consanguineous marriage between cousins from the paternal side increases the risk by 20% and from the maternal side by 50%. Similar reports of an increased odds ratio of 1.5-1.6x (50 to 60%) for diabetes among offspring of a consanguineous marriage between first-degree relatives have been published [15]. Other studies have shown that consanguinity increases the risk for T2DM by an earlier onset of the disease [8].

We found that parental consanguinity was not significantly associated with T1DM similar to other studies that have shown that the link between parental consanguinity and the development or risk for T1DM was not clearly defined [9]. Another similar study has shown that despite the high prevalence of familial T1DM among siblings, a positive family history of DM, and the presence of parental consanguinity, only one sibling was reported to have DM [7,16].

The story seems to be different with T2DM. The prevalence of DM was already reportedly high among offspring of mothers with DM (25.4%) and even higher among patients with consanguineous parents (38.5%) [17]. One of the studies suggested that the increased propensity of consanguineous marriage is a strong genetic component that exists in T2DM [18]. Genetic polymorphisms that influence the beta cells in insulin release and secretion such as gene *TCF7L2* locus rs7901695 and rs7903146, gene *KCNQ1* locus rs2237892, rs7756992, and gene *CDKAL1* locus rs7754840 and *KLF14* variant [19,20].

While there is a high prevailing prevalence of favored consanguineous marriage in Saudi Arabia, the level of education among females seemed to be inversely associated with consanguineous marriage [21-23]. Furthermore, the odds of a positive attitude towards consanguinity were shown to be 50% less among those who have received medical information and the rapid geographical urbanization on the effects of consanguinity and consanguineous marriages [24].

Limitations 

This study has some limitations. First, the family history of DM was not classified according to the type of DM. However, it is somewhat difficult for the subjects to determine what type of DM is in their relatives. Second, we did not include physical inactivity in the possible causative factors for DM. Nonetheless, T1DM is not closely related to physical inactivity and it's hard to measure activity precisely. Finally, BMI and age were not matched for the subjects and controls. In fact, it was difficult to match them as diabetic subjects tend to be more obese. On the other hand, in the regression analysis model where age and BMI were incorporated as independent factors, consanguinity remained associated with higher diabetes occurrence. 

## Conclusions

While there is a high prevalence of consanguineous marriage in the Saudi population, the association between consanguinity and the continued high prevalence of diabetes particularly type 2 diabetes has to be further elucidated. The risk of T2DM is increased in the presence of consanguinity by as much as 50%. The association between consanguinity up to first-degree relatives and the prevalence of T2DM is confirmed in this study. Ideas and perceptions about the relationship between DM and consanguinity in the general population are lacking. Henceforth, awareness campaigns to educate the public about consanguinity and its association with DM are recommended. This is a toss between a favored culture and the risk for inherited and genetically-linked diseases that poses threat to the health of the general population and the economy of the nation as well.
